# Effects of Alcohol and Cocaine in a Mutant Mouse Model of Predisposition to Post-Traumatic Stress Disorder

**DOI:** 10.3389/fphar.2020.00623

**Published:** 2020-05-08

**Authors:** Eleni Paizanis, Michela Crotti, Anthony Petit, Mathilde Règue, Virginie Beray-Berthat, Florence Noble, Laurence Lanfumey, Raymond Mongeau

**Affiliations:** ^1^ University of Caen Normandy, INSERM U1075 COMETE, Caen, France; ^2^ CNRS ERL 3649 «Pharmacologie et thérapies des addictions» Université Paris Descartes, Inserm UMR-S 1124, Paris, France; ^3^ IPNP-Université Paris Descartes, INSERM UMR-1266, Paris, France

**Keywords:** substance abuse, alcohol, cocaine, post-traumatic stress disorder, 5-HT_2C_ receptor editing, VGV mice, fear conditioning, fear extinction

## Abstract

Comorbidity between drug abuse and post-traumatic stress disorder (PTSD), a stress-related dysregulation of fear responses, is very high. While some drugs are known to increase fear and anxiety, there are only few data regarding interactions between voluntary drug consumption and fear memory. The spontaneous chronic consumption of either alcohol or cocaine under a 3-week free-choice progressive paradigm of alcohol (3/6/10%) or cocaine (0.2/0.4/0.6 mg/ml), was assessed in VGV transgenic mice, having full 5-HT_2C_ receptor editing and displaying PTSD-like behaviors. The consequences of these drug consumptions on the potentiated contextual and cued fear conditioning responses of VGV mice were assessed. The effects of drugs on hippocampal brain-derived neurotrophic factor (*Bdnf*) mRNA were measured as its expression was previously found to be decreased in VGV mice. Chronic alcohol consumption was similar in WT and VGV mice. In the alcohol condition, fear acquisition was not different at the end of the learning session and cue-fear extinction was facilitated. Regarding cocaine, in contrast to WT mice, VGV mice did not increase their drug consumption along with increasing doses, an effect that might be related with enhanced drug stimuli discrimination *via* increased 5-HT_2C_ receptors. Cocaine-intake VGV mice did not display the contextual fear generalization usually observed in control VGV mice. In addition, *Bdnf* expression was upregulated after either chronic alcohol or cocaine intake. Altogether, these results suggest that both chronic alcohol and cocaine voluntary oral consumptions can exert some therapeutic-like effects in a mutant model of PTSD predisposition.

## Introduction

Post-traumatic stress disorder (PTSD) is a stress-related disorder, characterized by hyperarousal, dysregulated context- and cue-induced fear responses, including fear memory extinction deficits and contextual fear generalization (Blechert et al., 2007; Lopresto et al., 2016). There is compelling evidence of a high comorbidity with substances dependence disorders (Bremner et al., 1996; Chilcoat and Menard, 2003). Indeed, prevalence of comorbid PTSD in alcohol misusers ranged from 2.0 to 63.0%, with most prevalence numbers between 15 and 30% (Debell et al., 2014). As with alcohol, cocaine abuse is often comorbid with PTSD, with a prevalence ranging from 8–23% to 23–43% of cocaine-dependent individuals meeting criteria for lifetime PTSD (Back et al., 2000; Najavits et al., 2003; Waldrop et al., 2007). Moreover, this comorbidity increases rates of substance craving and suicide attempts (McCauley et al., 2012). Cocaine users appear to have a hyperresponsiveness of the conditioned fear brain network (Kaag et al., 2016). While high co-occurrence of addiction and PTSD is certainly multifactorial, several hypotheses have been suggested (Chilcoat and Breslau, 1998): the *high-risk theory* states that substance abuse puts people at greater risk of experiencing traumatic events, and therefore addiction precedes PTSD onsets. The *susceptibility theory* suggests that substance abuse increase probability of developing PTSD after experiencing a traumatic event, by conferring a biological vulnerability to individuals. The *self-medication theory* states that PTSD patients use drugs to reduce distress, traumatic memories and associated symptoms. Finally, the *shared vulnerability theory* states that a genetic vulnerability underlies both PTSD risk and substance abuse.

There are few data regarding interactions between alcohol or cocaine and fear memories, which limit the development of specific therapeutic strategies for PTSD—addiction comorbidity. Animals more susceptible to traumatic-like stresses are more prone to cocaine addiction, compared to resilient subjects, probably because of an alteration in dopamine (DA) reuptake (Brodnik et al., 2017). Alcohol, like cocaine, acts through common neurotransmitter (DA of the VTA) but also through distinct neurobiological mechanisms, and may alter memory in multiple ways (Tipps et al., 2014). Beyond their rewarding effects, acute alcohol and cocaine trigger, respectively, anxiolytic effects mediated by activation of GABAergic receptors, and anxiogenic effects partly mediated by activation of corticotropin releasing factor (CRF) neurons (Ettenberg et al., 2015). 5-HT_2C_ receptors are known to be expressed by GABA interneurons and CRF neurons. Withdrawal anxiety triggered by chronic cocaine involves 5-HT_2C_ receptor activation and GABAergic neurotransmission within the raphe (Craige et al., 2005). Furthermore, the cocaine-induced enhancement of DA release within the ventral striatum is known to be regulated by 5-HT_2C_ receptors (Navailles et al., 2004). 5-HT_2C_ receptor pre-mRNA is also the target of post-transcriptional adenosine-to-inosine editing through adenosine deaminases, a process increased by alcohol consumption, and that occurs in conjunction with increased 5-HT_2C_ receptor expression (Watanabe et al., 2014).

We have established that VGV mice, that have only the fully edited VGV isoform of 5-HT_2C_ receptors, overexpress receptors because of a dysregulation of the truncated splicing variant (Martin et al., 2013). These mice exhibit decreased dopamine turnover (Olaghere da Silva et al., 2010) most likely because of the increased negative feedback regulation exerted by 5-HT_2C_ receptors on DA release (Millan et al., 1998). It has been argued that VGV mice might constitute a genetic model of PTSD predisposition based on several validity criteria (Règue et al., 2019): 1) Face validity: similarly to PTSD patients, VGV mice display aggressive interactions with conspecifics and are hyperaroused. A relatively brief aversive event can induce an extremely persistent fear memory in these animals. VGV mice, as PTSD patients, also display robust fear extinction deficits and fear generalization. 2) Predictive validity: treatments with paroxetine, approved for PTSD treatment, decreased the behavioral deficits of VGV mice. 3) Construct validity: similarly to victims of traumatic stresses, VGV mice have increased 5-HT_2C_ receptor neurotransmission. In particular, PTSD patients have exaggerated stress response to a 5-HT_2C_ agonist (Southwick et al., 1997) and display typical traits of serotonergic alterations including irritability, impulsivity and suicidability, which are themselves associated with 5-HT_2C_ receptor upregulation and altered 5-HT_2C_ mRNA splicing and editing (Niswender et al., 2001; Pandey et al., 2006; Di Narzo et al., 2014; Panagioti et al., 2015). Furthermore, as in patients, the VGV mice phenotype is associated with inflammation and hippocampal *Bdnf* mRNA expression reduction (Règue et al., 2019). In human, BDNF levels are determinant for fear extinction deficit and fear generalization (Mühlberger et al., 2014), and administration of alcohol and cocaine has been shown to alter brain BDNF in animal models (Li and Wolf, 2015; Stragier et al., 2015a; Stragier et al., 2015b).

Previous studies in humans and WT mice show that either alcohol or cocaine can have deleterious effects on fear memory extinction (Burke et al., 2006; Kaag et al., 2016; Scarlata et al., 2019). The main goal of the present study was not to assess, as in these previous studies, the effect of alcohol or cocaine *per se* on the basal phenotype, but rather to explore 1) whether the PTSD-like behavioral predisposition of VGV mice was associated with an increased propensity for voluntary alcohol or cocaine consumption, and 2) whether the robust WT versus VGV anxiety phenotype persisted in 3 experimental conditions: single house animals drinking either only water, water or alcohol and water or a cocaine solution, in a free choice paradigm. Furthermore, we assessed whether chronic exposure to these drugs alter hippocampal *Bdnf* expression together with the PTSD-like behavioral phenotype in the fear conditioning paradigm. Overall, contrary to our initial hypothesis, VGV mice do not have an enhanced voluntary drug consumption. Furthermore, and surprisingly, alcohol and cocaine exerted some therapeutic-like effects in the VGV vs WT mice paradigms aimed to explore either the fear extinction or the fear generalization deficits.

## Material and Methods

### Animals

Mice expressing VGV 5-HT_2C_ receptors generated and backcrossed for over 10 generations into the C57BL/6J genetic background and their control paired wild-type (WT) mice were used. Mice were kept under standard laboratory conditions (12 h light/dark cycle, room temperature 21 ± 1°C) with free access to food and water. They were housed one per cage to assess liquid consumption. All animals were 9–12 week-old at the beginning of each experiment and the experimental groups were randomly designed.

All procedures concerning animal care and treatment were carried out in accordance with the European Community Council Directive of September 22, 2010 (2010/63/UE) and the French regulations regarding the protection of animals used for experimental and other scientific purposes (D2013-118), with the approval of the Paris Descartes Animal Ethics Committee (#CEEA-34 Paris Descartes) and licensed by the Ministry of Education and Research.

### Free-Drug Consumption Paradigm

VGV vs WT mice (n = 8–16) were subjected to the free-choice ethanol or cocaine paradigm or water. For the “water” mice having access only to water, pipettes were filled with tap water.

For alcohol intake, the free-choice protocol paradigm was adapted from Kelaï et al. (2008). Each cage was equipped with four pipettes (10 ml), one containing tap water and the other three filled with ethanol diluted in water. Alcohol mice were exposed progressively to increasing concentrations of ethanol during 21 days (3% for 4, 6% for 4 and 10% for the subsequent 13 days). In these conditions, chronic ethanol intake leads to a maximum blood ethanol concentration of 50–70 mg per 100 ml during the drinking period (dark phase of the 24-h nycthemeral cycle: Murphy et al., 1986; Crabbe et al., 2009; Stragier et al., 2015a), a value far below that measured in human alcoholics (which can reach 300–600 mg per 100 ml). In rodents, unlike humans, self-administration only rarely ends with intoxication, ethanol intake stopping once blood concentration has reached ~50–70 mg per 100 ml, levels at which ethanol catabolism and elimination proceed at maximal rates in rodents (Stragier et al., 2015b). Absolute amounts of ethanol consumed and total fluid intake/kg body weight/24 h were determined for each mouse. Mice continued receiving 10% ethanol in their home cage during behavioral experiments period.

For cocaine intake, mice had access to two 10 ml pipettes, one containing water, the other containing cocaine diluted in tap water, at increasing concentrations (0.2/0.4/0.6 mg/ml). Each concentration was available for 4 days separated by a 3-day wash-out period to avoid the anorexigenic effect of cocaine. Cocaine 0.6 mg/ml was reintroduced in the home cages one day before fear conditioning. Absolute amounts of cocaine (as mg) consumed and total fluid intake/kg body weight/24 h were determined for each mouse.

### Subchronic Parenteral Exposure to Alcohol and Cocaine

In these experiments, non-contingent modes of administration were used to assess in WT mice if alcohol and cocaine had effects consistent with those of the literature.

Intraperitoneal subchronic alcohol injections: A 25% (vol/vol) ethanol solution in 0.9% saline was administered i.p. at 10 ml/kg. Mice received injections of either saline or 2 g/kg ethanol once daily for five consecutive days (see **Supplemental Figure 2A**). After a 2-day drug-free period mice were behaviorally tested on the third day in absence of alcohol. Fear conditioning experiments were performed with 8–12 mice per group.

Intraperitoneal subchronic cocaine injections: After a 5-day acclimation period, i.p. injections at 10 ml/kg injections of saline or cocaine (20 mg/kg) in 0.9% saline was done once daily for five consecutive days (**Supplemental Figure 2B**). After two drug-free days mice were behaviorally tested on the third day in the absence of drug. Fear conditioning experiments were performed with 8–12 mice per group.

### Fear Conditioning

After chronic or subchronic paradigms (see **Figures 3 A–C**, and **Supplemental Figures S2A, S3A**), mice (n = 8–16) were assessed in the fear conditioning paradigm, consisting of three consecutive days of behavioral testing, which was conducted in a chamber (26 × 18 × 22 cm), housed in a sound-attenuating box, with aluminum sidewalls, Plexiglas rear and front walls, and a stainless steel grid floor (MED Associates, St. Albans, VT, USA). On day-1 (fear conditioning), mice were individually placed into the dark conditioning chamber odorized with 1% carvone (mint odor) and allowed to acclimate for 3 min (baseline). Then, animals received six times an association between an auditory conditional stimulus (CS; 30 s, 2.5 kHz, 85 dB) immediately followed by the unconditioned stimulus [US; 2 s, 0.5 mA foot-shock, inter-trial intervals (ITI) of 2  min]. On day-2 (contextual fear test), mice were returned to the same conditioning chamber, odorized with mint, and allowed to explore the cage 3  min without presentation of CS or US. The third day (cued extinction), mice were placed in chambers with modified context odorized 0.5% isopentyl acetate (banana odor) and different cage walls. After measuring baseline freezing (which may indicate contextual fear generalization), the CS alone was presented 15 times, separated by 5 s ITIs. The extinction index was defined by the maximal CS-induced freezing at the beginning of the session minus that at the end of the session. All sessions were recorded using infrared cameras and controlled by a computerized system interface (MED Associates). Freezing behavior, defined as complete absence of movements except for respiration, was measured every 1 s using the Videofreeze software (MED Associates, St. Albans, USA) and expressed as percentage time spent freezing. Immediately after tone extinction, hippocampi were collected for gene expression studies.

### Gene Expression

For quantification of total *Bdnf* mRNA expression of the different groups (n = 4–12), hippocampi were quickly removed and frozen in liquid nitrogen. Total mRNA was extracted using TRI Reagent (Ambion, Applied Biosystems, Courtaboeuf, France), following manufacturer’s instructions. Reverse-transcription was performed using High Capacity cDNA Reverse Transcription kit and PCR amplifications were performed using a SYBR Green mix (KAPA SYBR Fast qPCR Master Mix, KAPA Biosystems, MA, USA) and specific primers for total *Bdnf*, forward: 5′-TGCAGGGGCATAGACAAAAG-3′, reverse: 5′-TGAATCGCCAGCCAATTCTC-3′; and *Actb* reference gene; forward: 5′-CCACCATGTACCCAGGCATT-3′, reverse: 5′-CGGACTCATCGTACTCCTGC-3′. (For detailed cycling protocols see Règue et al., 2019). Gene expression was normalized by reference to the housekeeping gene β-actin and analyzed using the 2ΔΔCT method (Delta-Delta Comparative Threshold; Livak and Schmittgen, 2001).

### Statistical Analysis

Data presented as mean  ±  S.E.M. were analyzed using Prism (GraphPad, San Diego, USA). For two group comparisons, an unpaired Student’s t-test was used, with Welch’s correction if needed. All remaining data were compared using a two-way analysis of variance (ANOVA), with repeated measures followed when necessary, by a Bonferroni post-hoc test or a one-way ANOVA analysis with repeated measures followed by a Dunnett post-hoc test. qRT-PCR results were analyzed with a two-group comparison, as the 2ΔΔCt method generates groups that are not independent. Outliers were identified using a z-score cut-off and were excluded from the statistical analysis (two in water VGV group, one both in WT and VGV ethanol groups, one in VGV cocaine group) and some video files (7) for day-1 fear conditioning were lost due to a technical problem. Statistical significance was set at p <0.05.

## Results

### Evolution of Alcohol and Cocaine Consumption in VGV Mice

**Figure 1:**
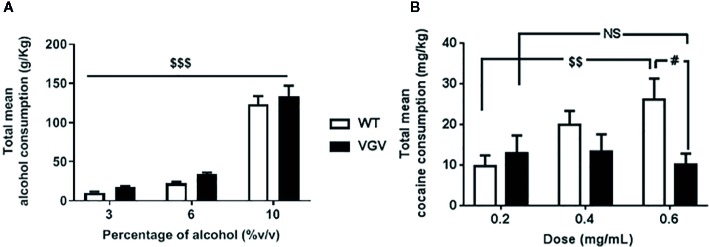
Evolution of alcohol and cocaine consumption in VGV mice. Total free-choice consumption of alcohol **(A)** or cocaine **(B)** in VGV and WT mice in a 3-week-progressive drug treatment. **(A)** ANOVA analysis with repeated measures showed a main effect of alcohol concentration [F(2,60) = 163.4, p < 0.001] but no effect of genotype [F(1,30) = 1.97, p = 0.17] and no interaction [F(2,60) = 0.06, p = 0.94]. **(B)** There was a significant interaction genotype × cocaine-dose effect [F(2,36) = 3.46, p <0.05], but no effect of genotype [F(1,18) = 2.87, p = 0.11] or of cocaine concentration [F(2,36) = 1.95, p = 0.16]. The mean consumption of cocaine did not increase in VGV mice with the increasing dose contrary to WT mice (p < 0.01), and for the highest dose (0.6 mg/ml) VGV mice exhibited almost ~60% less consumption compared to WT mice (WT vs VGV, p < 0.05). Data are expressed as mean ± S.E.M. VGV vs WT mice, (alcohol-treated WT and alcohol-treated VGV mice, n = 16; cocaine-treated WT mice, n = 8; cocaine-treated VGV mice, n = 12, ^#^p < 0.05, effect of drug concentration, ^$$^p <0.01, ^$$$^p <0.001). NS, non significant.

We assessed the propensity of WT and VGV mice to consume alcohol or cocaine. Regarding alcohol intake, we observed during a 3-week free-choice exposure that the mean ethanol intake progressively increased along with percentage of alcohol in both WT and VGV mice (**Figure 1A**). ANOVA analysis with repeated measures showed a main effect of alcohol concentration [F(2,60) = 163.4, p <0.001] but no effect of genotype [F(1,30) = 1.97, p = 0.17] and no interaction [F(2,60) = 0.06, p = 0.94].

Regarding cocaine, the evolution of consumption was different depending on the genotype (**Figure 1B**). There was a significant genotype x cocaine-dose interaction, F(2,36) = 3.46, p <0.05, but no effect of cocaine concentration, F(2,36) = 1.95, p = 0.16 or effect of genotype F(1,18) = 2.87, p = 0.11. Statistical analysis revealed that, in contrast to WT mice, for whom cocaine intake increased along with increasing concentrations (0.2 vs 0.6%, p <0.01), the mean consumption of cocaine did not rise in VGV mice. This was also suggested by the significant difference (~60%) observed between the two genotypes for the highest dose 0.6% (WT vs VGV, p <0.05).

### Both Chronic Self-Administered Alcohol and Cocaine Altered VGV Mice Fear Behaviors

**Figure 2:**
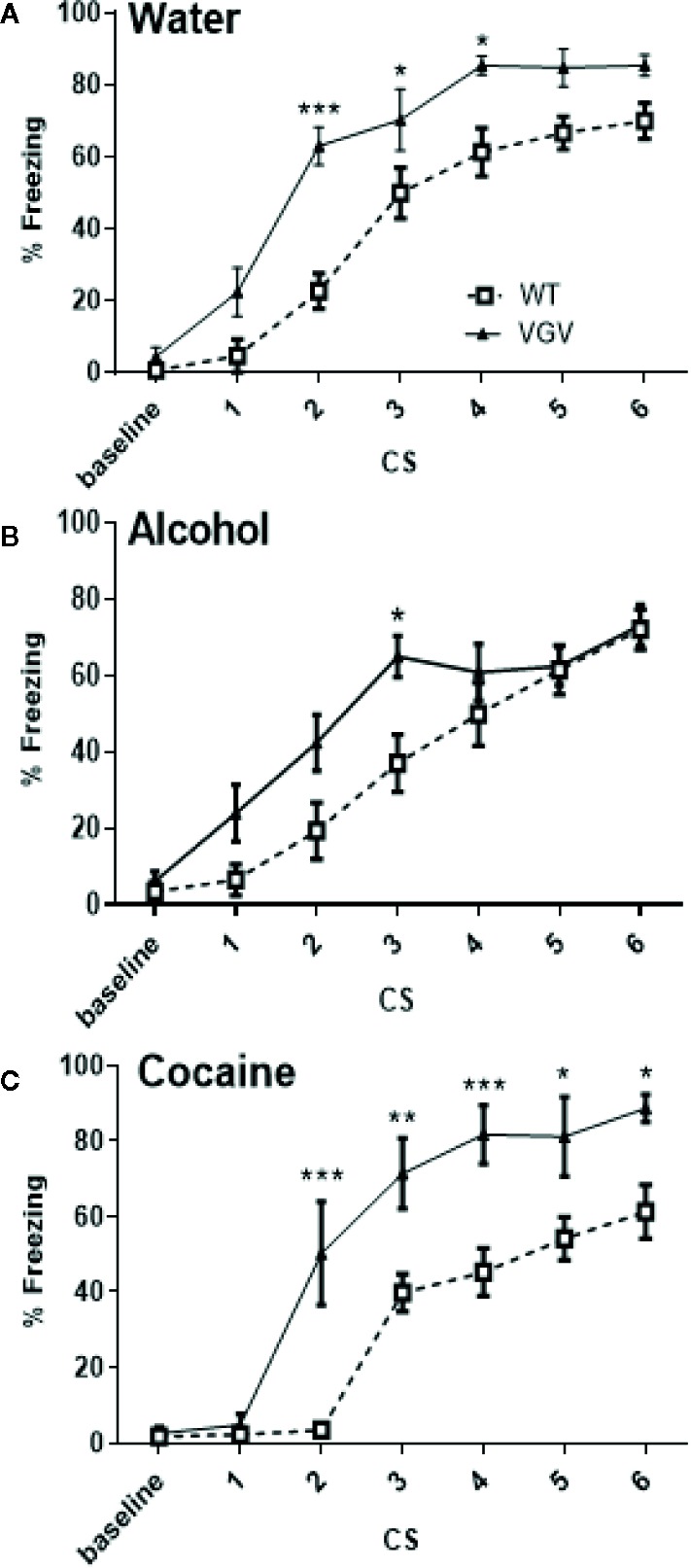
Self-administered chronic alcohol, but not chronic cocaine, reduced fear acquisition in VGV mice. **(A)** Water-treated VGV mice acquired fear conditioning faster compared to water-treated WT mice. Two-way ANOVA with repeated measures indicated a significant effect of genotype [F(1,19) = 17.11, p <0.001], an interaction time of CS × genotype [F(6,114) = 3.27, p <0.01], and a time effect [F(6,114) = 103.3, p <0.001]. **(B)** Alcohol-treated WT and VGV groups were different [genotype effect F(1,23) = 4.18, p <0.05, time effect F(6,138) = 49.09, p <0.001, interaction CS × Genotype F(6,138) = 2.36, p <0.05]. VGV mice are different from WT mice only at CS 3. **(C)** In the cocaine condition, the large difference between VGV and WT remained [genotype effect F(1,108) = 24.26, p <0.001, time effect F(6,108) = 62.08, p <0.001, interaction CS × genotype F(6,108) = 4.77, p <0.001]. *p <0.05; **p <0.01, ***p <0.001. Data are expressed as mean ± S.E.M.,VGV vs WT mice, (water-treated WT, n = 10, or VGV mice, n = 11; alcohol-treated WT, n = 12, or VGV mice, n = 13; cocaine-treated WT mice, n = 8; cocaine-treated VGV mice, n = 12; *p <0.05; ***p < 0.001).

On day-1, as expected from our previous study (Règue et al., 2019), isolated VGV mice receiving only water acquired fear conditioning faster compared to WT mice in the same conditions (**Figure 2A**). The global two-way ANOVA analysis with repeated measures, including all groups [3 × 2; **Figure 2**; showed a significant group × time interaction (F30,360) = 2.82, p <0.001), a time effect (F6,360) = 195.6, p <0.001) and a group effect (F5,60) = 9.47, p <0.001]. We have then examined the genotype effect in each of the three conditions, WT and VGV mice exposed to water (**Figure 2A**), alcohol (**Figure 2B**) or cocaine (**Figure 2C**). In the water condition, the two-way ANOVA indicated a significant effect of genotype (F(1,19) = 17.11, p <0.001), an interaction time of CS × genotype [F(6,114) = 3.27, p <0.01], and a time effect [F(6,114) = 103.3, p <0.001] and a post-hoc analysis showed a significant difference at CS2, CS3 and CS4, while the difference tended to be significant at CS5 and CS6. A similar difference was observed between WT and VGV mice having cocaine intake, from CS2–CS6 (**Figure 2C**), [genotype effect F(1,108) = 24.26, p <0.001, time effect F(6,108) = 62.08, p <0.001, interaction CS x genotype F(6,108) = 4.77, p <0.001]. However, in the alcohol condition VGV mice compared to WT mice were different for the third CS-US association only (**Figure 2B**), [genotype effect F(1,23) = 4.18, p <0.05, time effect F(6,138) = 49.09, p <0.001, interaction CS × genotype F(6,138) = 2.36, p <0.05].

**Figure 3:**
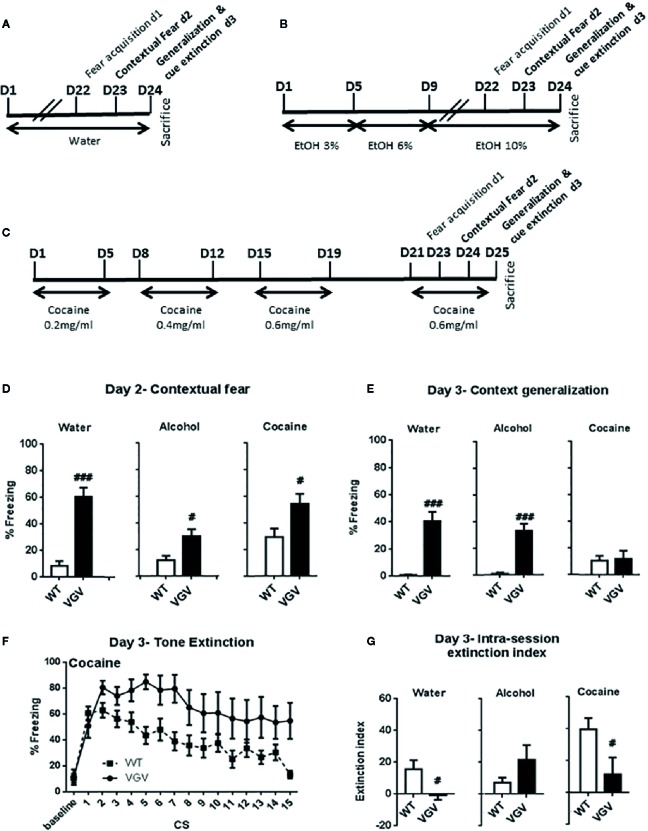
Both self-administered chronic alcohol and cocaine altered fear behaviors in VGV mice **(A–C)** Protocol of water, alcohol or cocaine intake before and during the conditioned fear testing period. **(D)** During the contextual fear test VGV mice presented higher freezing than WT mice [*t*_(19)_ = 6.27, *p <* 0.001, *t*_(23)_ = 2.65, *p <* 0.007, *t*_(18)_ = 2.53, *p* = 0.011, for water (WT, n = 10, VGV, n = 11) alcohol (WT, n = 12, VGV, n = 13) and cocaine (WT, n = 12, VGV, n = 8), respectively] **(E)** Water- and alcohol-treated VGV mice displayed higher fear generalization during the baseline period of the tone session [*t*_(23)_ = 6.06, *p <* 0.001, *t*_(30)_ = 6.19, *p <* 0.001, respectively], but not cocaine-treated mice compared to similarly treated WT mice. In addition, in the cocaine condition there was a small amount of fear generalization in WT animals (one sample t-test compared to 0; t_11_ = 3.06; *p <*0.05). **(F)** Curve extinction profile of WT and VGV mice treated with cocaine. A two-way ANOVA analysis between WT and VGV cocaine-treated mice revealed a genotype effect [F(1,18) = 8.15, p <0.05], an effect of time [F(14,252) = 5.92, p <0.001] and an interaction CS × genotype [F(14,252) = 1.80, p <0.05]. **(G)** Water- and cocaine-treated VGV mice showed reduced fear extinction index (defined as [mean freezing at CS1 and CS2] − [mean freezing at CS14 and 15]) compared to similarly treated WT mice [water: *t*_(23)_ = 2.52, *p* = 0.0096; cocaine: *t*_(18)_ = 2.37, *p* = 0.0014], but VGV mice were not different from WT mice in the alcohol treatment condition. Data are expressed as mean ± S.E.M. VGV vs WT mice (water-treated WT, n = 13, or VGV mice, n = 12; alcohol-treated WT, n = 16, or VGV mice, n = 16; cocaine-treated WT mice, n = 8; cocaine-treated VGV mice, n = 12; ^#^p < 0.05; ^###^p < 0.001).

On day-2, as expected (see Règue et al., 2019), water-treated VGV mice presented high freezing during the contextual fear test, while WT mice had minimal freezing in the present experimental conditions (**Figure 3D**; delta = 51.89 ± 7.12%, *t*_(19)_ = 6.27, p <0.001). When comparing the effects of the different treatments, a two-way ANOVA analysis showed a significant genotype × treatment interaction (F2,60) = 4.88, p <0.05), a genotype effect (F1,60) = 44.16, p <0.001) and a treatment effect (F2,60) = 7.03, p <0.01). In more details, there was a smaller but significant difference in contextual fear between WT and VGV mice in the alcohol condition (**Figure 3D**; delta = 17.7 ± 5.7%, *t*_(23)_ = 2.65, *p* = 0.007). Furthermore, the WT vs VGV difference (*t*_(18)_ = 2.53, p = 0.011) existed in the cocaine intake condition (**Figure 3D**).

On day-3, as expected from our previous study (Règue et al., 2019), at baseline, water-treated VGV mice exhibited contextual fear generalization in the new context before the tone extinction session (*t*_(23)_ = 6.06, *p <*0.001), and alcohol-treatment did not alter this difference (*t*_(30)_ = 6.19, *p <*0.001, **Figure 3E**). Indeed, the two-way ANOVA analysis showed a significant genotype × treatment interaction (F2,71) = 9.71, p <0.001), and a genotype effect (F1,71) = 47.65, p <0.001). However, in the cocaine condition, VGV mice did not display contextual fear generalization [(t_(18_) = 0.20, p = 0.42, **Figure 3E**].

During the repeated tone exposure, VGV mice exhibited a higher freezing throughout the session compared WT mice in all the conditions (**Supplemental Figure S1** and **Figure 3F**). The global two-way ANOVA analysis with repeated measures, including all groups, revealed a group × time interaction [(F70, 994) = 1.85, p <0.001], a time effect [(F14, 994) = 13.19, p <0.001] and a group effect [(F5,71) = 45.52, p <0.001].

VGV vs WT mice were further analyzed for differences along the fear extinction test. The two-way ANOVA analysis in cocaine-treated mice revealed a genotype effect [F(1,18) = 8.15, p <0.05], an effect of time of CS [F(14,252) = 5.92, p <0.001] and an interaction time of CS × genotype [F(14,252) = 1.80, p <0.05]. However, post-hoc analysis indicated a significant difference only at CS5 (p <0.01) in the cocaine condition (**Figure 3F**). Observation of the curve profile indicated some apparent extinction in both WT and VGV mice treated with cocaine (**Figure 3F**). Nevertheless, in cocaine-treated VGV mice, the decrease of CS-induced freezing between the second CS of the session (80.5 ± 5.2%) and the last (54.8 ± 13.9%) failed to reach statistical significance (p = 0.12, Student t-test). Regarding the fear extinction index (**Figure 3G**), the two-way ANOVA analysis showed a significant genotype x treatment interaction [(F2, 71) = 5.39, p <0.01]. Cocaine-treated VGV mice showed a reduction of the fear extinction index compared to similarly treated WT mice, as usually observed in the control condition [water: *t*_(23)_ = 2.52, *p* = 0.0096; cocaine: *t*_(18)_ = 2.37, *p* = 0.0014].

In contrast, alcohol-treated VGV mice did not show the fear extinction deficit as normally observed in water-treated VGV mice (**Figure 3G**; **Supplemental Figure S1**). However, although the amount of freezing in VGV mice treated with alcohol remained high during the fear extinction procedure, there was a significant decrease of CS-induced freezing between the second CS of the session (80.1% ± 6.4) and the last (53.6% ± 8.7, p <0.05, Student t-test).

### Both Self-Administered Chronic Alcohol and Cocaine Altered Hippocampal Bdnf mRNA Expression in VGV Mice

**Figure 4:**
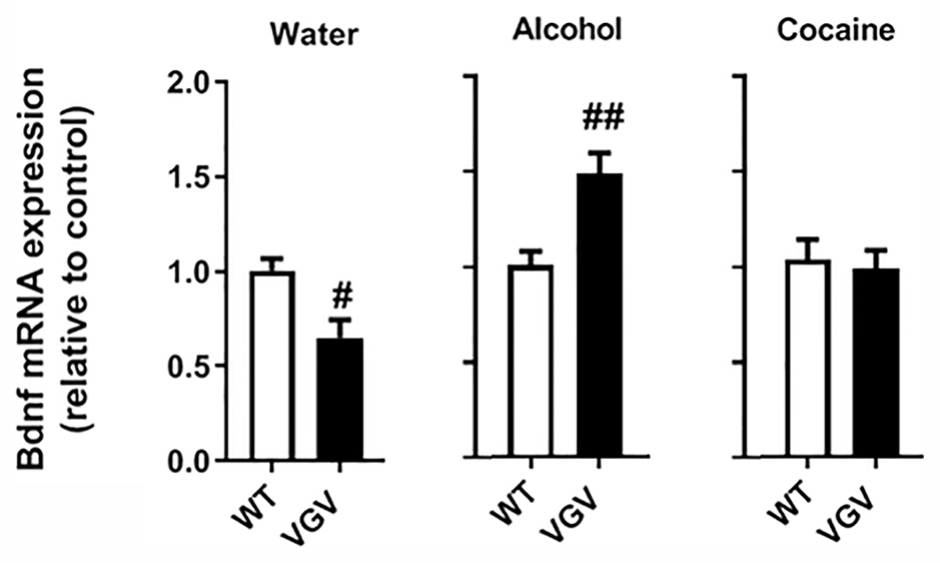
Self-administered chronic alcohol and cocaine altered hippocampal *Bdnf* mRNA expression in VGV mice. *Bdnf* mRNA expression was decreased in VGV mice compared to WT mice (*t*_(6)_ = 3.01, *p <*0.05). Alcohol-treated mice exhibited a ~1.5 fold increase compared to alcohol-treated WT mice (*t*_(9)_ = 3.51, *p <*0.01). In the cocaine condition, VGV mice did not differ from WT mice. Data are mean expression levels ± S.E.M. normalized to WT mice. VGV vs WT mice (water-treated WT or water treated VGV mice, n = 4; alcohol-treated or alcohol-treated VGV mice, n = 5–6; cocaine-treated WT mice, n = 8; cocaine-treated VGV mice, n = 12, ^#^p <0.05; ^##^p <0.01).

It was previously reported that hippocampal *Bdnf* mRNA expression is reduced in VGV mice compared to WT mice (Règue et al., 2019). In the water-intake condition, *Bdnf* expression was decreased in the hippocampus of VGV mice (*p <*0.05; **Figure 4**). Alcohol-intake VGV mice exhibited a ~1.5 fold increase compared to alcohol-intake WT mice (*p <*0.01; **Figure 4**). In turn, there was no alteration of hippocampal *Bdnf* levels of VGV compared to WT mice in the cocaine condition (**Figure 4**).

### Subchronic Exposure to Either Alcohol or Cocaine Have Anxiogenic Effects in the Fear Conditioning Paradigm in C57BL/6J WT Mice

In view of the rather surprising therapeutic-like effects of drugs in VGV mice, we assessed the effect of non-contingent, rather than voluntary, alcohol and cocaine administration in WT C57BL/6J mice. We used here a more classical mode of administration, that is five successive i.p. injections followed by a 2-day withdrawal period before fear conditioning testing period (**Supplemental Figures S2A** and **S3A**).

Conditioned fear acquisition was not altered by alcohol injection (**Supplemental Figure S2B**). The following day, however, ethanol-treated mice exhibited increased total freezing throughout the contextual fear test session compared to saline-treated mice (**Supplemental Figure S2C**). Nevertheless, on day 3, saline-treated and alcohol-treated mice did not significantly differ in the conditioned tone extinction test (**Supplemental Figures S2D, E**).

Conditioned fear acquisition was not altered by the cocaine injection (**Supplemental Figure S3B**). The following day, here again, the cocaine-treated mice exhibited increased total freezing during the contextual fear test session compared to saline-treated mice (**Supplemental Figure S3C**). Furthermore, on Day 3, cocaine-treated mice showed a significant reduction of extinction compared to saline-treated mice (**Supplemental Figures S3D, E**).

## Discussion

In line with the shared vulnerability theory of addiction, it was hypothesized that since VGV mutant mice is a valid predisposition model of PTSD-like behaviors (Règue et al., 2019), it might equally be a model of alcohol and cocaine abuse. Overall, the results of the present study do not support this notion in a voluntary alcohol and cocaine administration paradigm. VGV mice started consuming more alcohol at low doses, but this profile was not confirmed at a higher dose, while their cocaine consumption did not escalate by increasing pipette drug concentration. It is not excluded however, that these transgenic mice display addiction-like behaviors in other conditions (following a prior stress, in conditioned place preference or intravenous auto-administration paradigms). Furthermore, in view of their conditioned fear extinction deficit, VGV mice may similarly have a conditioned drug extinction deficit. Relapse in VGV mice should be investigated in futures studies despite they do not consume more drugs.

Considering that VGV mice are more vulnerable to anxiogenic stimuli (Martin et al., 2013), it may be argued that VGV mice limited their cocaine consumption because of their sensitivity to the interoceptive anxiogenic-cues associated with cocaine administration. From a neurotransmission point of view, it has also been shown that accumbal 5-HT_2C_ receptors regulate the discriminative stimulus effects of cocaine (Filip and Cunningham, 2002). The increased expression of 5-HT_2C_ receptors in VGV mice might thus limit consumption of cocaine, as the aversive interoceptive cues were more intense in these animals. This is in line with the study of Rocha and colleagues (2002) showing a higher reinforcing effect of cocaine in mutant mice devoid of 5-HT_2C_ receptors.

The goal of the present study was to determine if the robust fear phenotype of VGV vs WT mice remains in the context of an ongoing chronic alcohol or chronic cocaine consumption, rather than assessing the effect of drug administrations in baseline conditions, as it was done in numerous previous studies (Burke et al., 2006; Wood et al., 2007; Skelly et al., 2015; Scarlata et al., 2019). The reason for choosing voluntary drug consumption, rather than i.p. or i.v. catheter injections, was driven by the fact that VGV mice were more sensitive to innate fear (animal handling) and inflammation (Martin et al., 2013; Règue et al., 2019), two side effects avoided in the free choice drinking conditions. One limitation of this however, is that the final amount of drug received is not necessarily equal between genotypes, which might confound the effect of drugs on fear. Nevertheless, the surprising result that clearly emerges from the present study is that, although no genotype effect was detected in drug intake, both chronic alcohol and cocaine voluntary oral consumption can exert some therapeutic-like effects in VGV compared to WT mice. In view of these results we decided to investigate in WT mice the effects of alcohol and cocaine in conditions often reported in the literature (subchronic i.p. administration).

In contrast to one’s assumption about anxiety disorders, GABAergic anxiolytic drugs such as benzodiazepines and alcohol are to be avoided in PTSD (Guina et al., 2016). Several preclinical studies have shown they might exacerbate fear extinction deficits (Holmes et al., 2012; Scarlata et al., 2019). We found that subchronic alcohol, followed by withdrawal, increases contextual fear in WT mice, although it did not have here a significant effect on cued fear extinction. In contrast, in VGV mice having an anxiety phenotype, free choice alcohol may have had an anxiolytic-like effect. This was apparent at the end of the fear conditioning process and from the reduced difference in contextual fear compared to WT mice that had free choice alcohol. This would need to be further investigated in VGV mice, treated or not with alcohol, although an anxiolytic effect would not be surprising in view of the known effects of alcohol in trait anxious animals (Sharko et al., 2016). Furthermore, and interestingly, alcohol abolished the fear extinction deficit normally observed in VGV mice. This is consistent with the self-medication theory of addiction: PTSD patients might be inclined to increase their alcohol consumption to obtain temporary anxiolysis. Nevertheless, anxiolytic drugs might have a deleterious effect on fear extinction memory depending on the time, the context and the path of administration, which necessarily complicate their clinical use (Hart et al., 2014).

As expected from the literature (Burke et al., 2006), we found deleterious effects of cocaine i.p. administration in WT mice, since it increased contextual fear and generated a fear extinction deficit. Although the fear extinction deficit still existed in VGV compared to WT mice after chronic cocaine, chronic voluntary cocaine reduced their contextual fear generalization compared to WT mice. Note that in basal conditions, WT C57BL6/J mice do not show any significant contextual fear generalization. This is remarkable, since in our previous studies (Règue et al., 2019), chronic oral treatment with one of the two drugs approved for the treatment of PTSD, paroxetine, did have an anxiolytic effect in VGV mice, but failed to abolish fear generalization. The decrease of contextual fear generalization by chronic cocaine may be surprising if one considers that cocaine is an anxiogenic drug and that generalization is a form of fear sensitization. Nevertheless, increased dopamine acting on D_2_ receptors, through cocaine-induced DA reuptake inhibition within the frontal cortex or the extended amygdala may explain this effect (De Bundel et al., 2016; Lee et al., 2016). Indeed, increased DA neurons activity was shown to prevent fear sensitization, as the DA released in conjunction with a CS is crucial in determining threat discrimination and uncertainty about a set of stimuli predicting an aversive stimulus (Jo et al., 2018). Our results thus suggest that fear sensitization in VGV mice result from the deleterious effect of increased 5-HT_2C_ receptor-mediated inhibition of DA release and that drugs increasing DA concentration may be therapeutic for fear sensitization disorders.

Furthermore, similarly to what has been found with numerous antidepressants (Castren et al., 2007; Règue et al., 2019), chronic cocaine increased hippocampal *Bdnf* expression. This is also the case for novel selective triple reuptake inhibitors that block, like cocaine, DA in addition to serotonin and noradrenaline reuptake carriers (Larsen et al., 2007). The normalization of hippocampal *Bdnf* may be involved in restoring normal context discrimination after cocaine consumption in VGV mice. Nevertheless, alcohol consumption increased hippocampal *Bdnf* in VGV mice compared to WT mice, facilitated fear extinction, but this treatment failed to normalize context generalization. It might be argued that both changes in DA transmission and *Bdnf* expression are necessary to restore context discrimination. Previous studies from our group have shown that the increase in *Bdnf* gene transcription after chronic voluntary ethanol intake is probably the result of epigenetic modifications, in particular *Hdacs* expression and *Bdnf* chromatin remodeling (Stragier et al., 2015b). Furthermore, note that in other conditions of alcohol administration, corresponding to greater levels of intoxication, a decrease *Bdnf* expression was observed, and that an increase expression of *Bdnf* might serve as an adaptation preventing excessive consumption (Haun et al., 2018).

In conclusion, contrary to our initial hypothesis, VGV mice do not appear to be a model of increased alcohol or cocaine consumption, at least in the present conditions of contingent drug administration. Instead and surprisingly, therapeutic-like effects of these drugs on the PTSD-like phenotype of VGV mice were observed. In contrast, in WT mice, subchronic non-contingent administrations of these drugs produced aversive effects. These data further support that VGV mice might be useful to investigate therapeutic vs addictive drugs windows and for innovative comorbidity treatment discovery.

## Data Availability Statement

The datasets generated for this study are available on request to the corresponding author.

## Ethics Statement

The animal study was reviewed and approved by Paris Descartes Animal Ethics Committee (#CEEA-34 Paris Descartes) and licensed by the Ministry of Education and Research.

## Author Contributions

RM, LL and EP: All aspects of the study. FN and VB-B: Writing. MC, AP, and MR: Experiments.

## Funding

This work was supported by grants FRA 2018/11 and FRA 2017/18.

## Conflict of Interest

The authors declare that the research was conducted in the absence of any commercial or financial relationships that could be construed as a potential conflict of interest.
