# Supplementary material for: Effects of Alcohol and Cocaine in a Mutant Mouse Model of Predisposition to Post-Traumatic Stress Disorder
Source: Front Pharmacol. 2020 May 8;11:623. doi: 10.3389/fphar.2020.00623 (PMC7232567; doi:10.3389/fphar.2020.00623)
Supplement: Supplementary file 1 [file DataSheet_1.pdf]

Supplemental materials

1) Expression of conditioned fear behaviors in WT and VGV mice

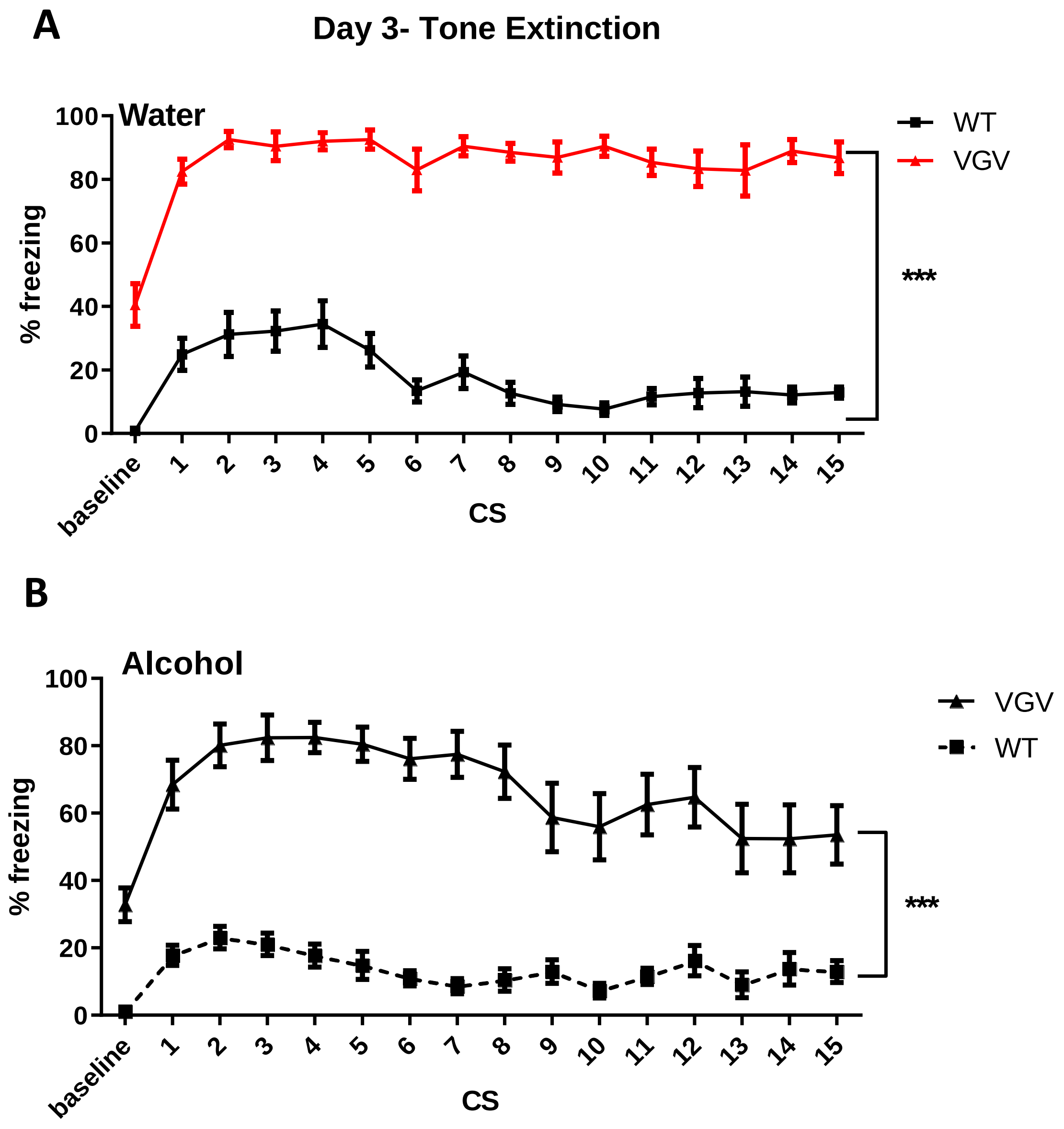

Figure S1: Fear extinction profile of WT vs VGV mice exposed to drinking pipettes
A. Curve profile of freezing behavior during the tone extinction session of WT and VGV mice that consumed water. A two-way ANOVA analysis between WT and VGV water-treated mice revealed a genotype effect [F(1,23)=342.6, p<0.001], an effect of time [F(14,322)=4.36, p<0.001] and an interaction CS x Genotype [F(14,322)=2.10, p<0.05]. VGV vs WT mice for every CS, *** p<0.001, WT, n=13, VGV, n=11. **B.** Curve profile of freezing behavior during the tone extinction session of WT and VGV mice that consumed alcohol. A two-way ANOVA analysis between WT and VGV alcohol-treated mice revealed a genotype effect [F(1,30)=76.75, p<0.001], an effect of time [F(14,420)=5.05, p<0.001] and an interaction CS x Genotype [F(1,420)=2.42, p<0.001]. VGV vs WT mice for every CS, *** p<0.001, n=16 per group.

2) Effects of subchronic alcohol on fear conditioning behaviors

Intraperitoneal subchronic alcohol treatments

The protocol was adapted from Quiñones and colleagues (Psychopharmacology 2015 232:3615-22). A 25 % (vol/vol) ethanol solution was prepared by diluting a 96 % ethanol stock solution with 0.9 % saline and administered via intraperitoneal (i.p.) injections at 10 ml/kg. After a 5-day acclimation period, mice received injections of either saline or 2 g/kg ethanol once daily for 5 consecutive days followed by a 2-day wash-out period. Blood-ethanol concentrations have been shown to peak at around 150 mg/dl 30 min after i.p. injection (Beaudet et al, 2016; Alcohol. Clin. Exp. Res. 40:2591). After a 2-day drug-free period mice were behaviorally tested on the third day in the absence of drug. Experiments were performed with 8-10 mice per group.

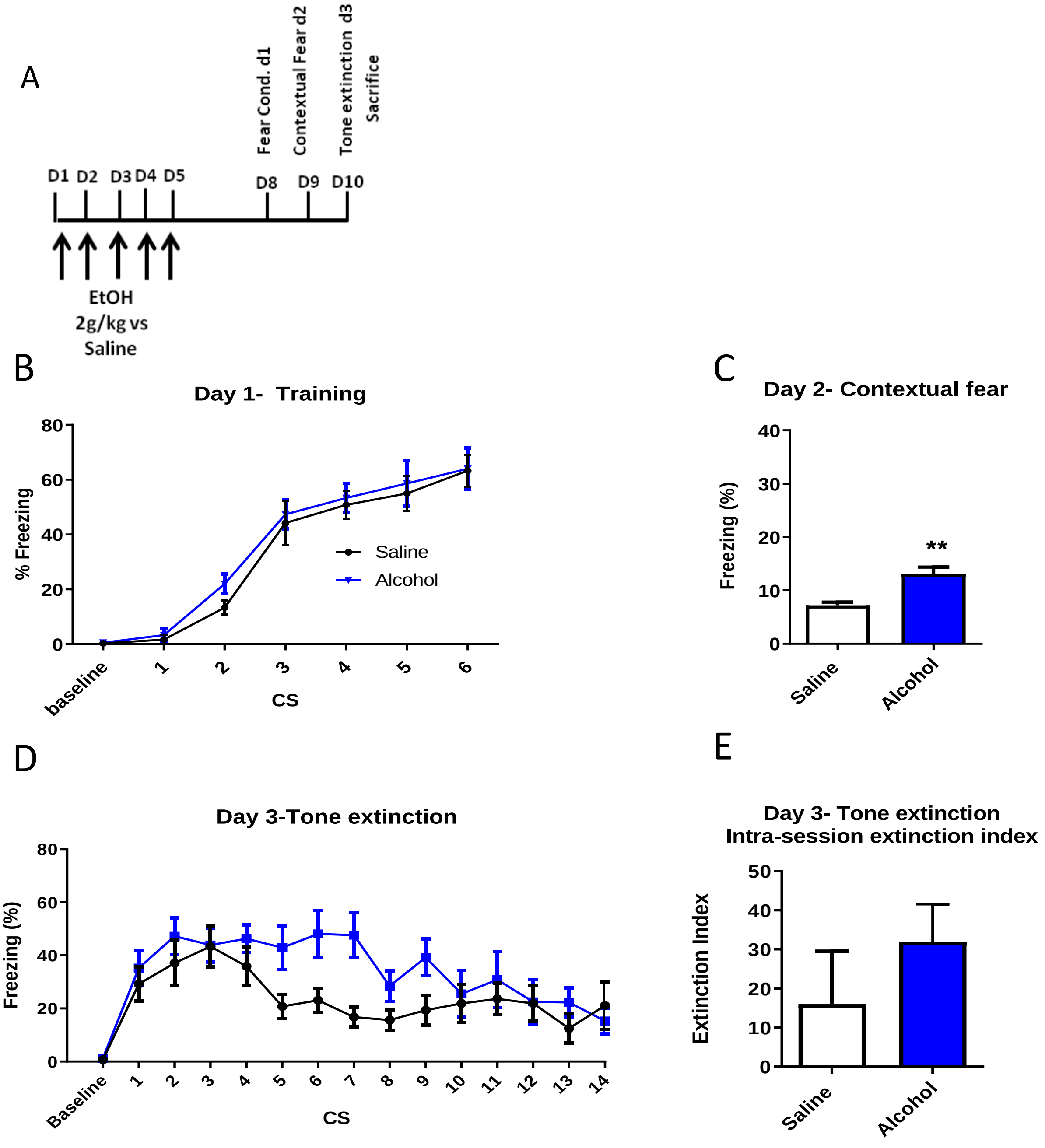

Figure S2: Subchronic exposure to alcohol increased contextual fear in C57BL/6J mice. A. Experimental design**. B.** There was no effect of alcohol on fear acquisition in WT C57BL/6J mice. C. The following day, ethanol-treated mice exhibited increased total freezing throughout contextual fear session compared to saline-treated mice [*t*(38) =4.33, *p* <0.01]. D. On Day 3, when mice were exposed to a different context, saline- and alcohol-treated mice displayed conditioned cue extinction shown by a CS effect [two-way ANOVA F(13,208)=4,31, p<0.001] but no effect of genotype, [F(1,16)=3.16, p=0.09], and no interaction, [F(13,208)=1.54, p=0.10]. E. Moreover the extinction index, defined as (Day 3 freezing at CS2) − (Day 3 freezing at CS14) was similar between groups. Alcohol (n=10) vs saline (n=8) **p<0.01.

3) Effects of subchronic cocaine on fear conditioning behaviors

Intraperitoneal subchronic cocaine treatment

After a 5-day acclimation period, i.p. injections at 10 ml/kg injections of saline or cocaine HCL (20 mg/kg) dissolved in 0.9% saline was done once daily for 5 consecutive days followed by 2-day wash-out period. After 2 drug-free days mice were behaviorally tested on the third day in the absence of drug. Experiments were performed with 12 mice per group.

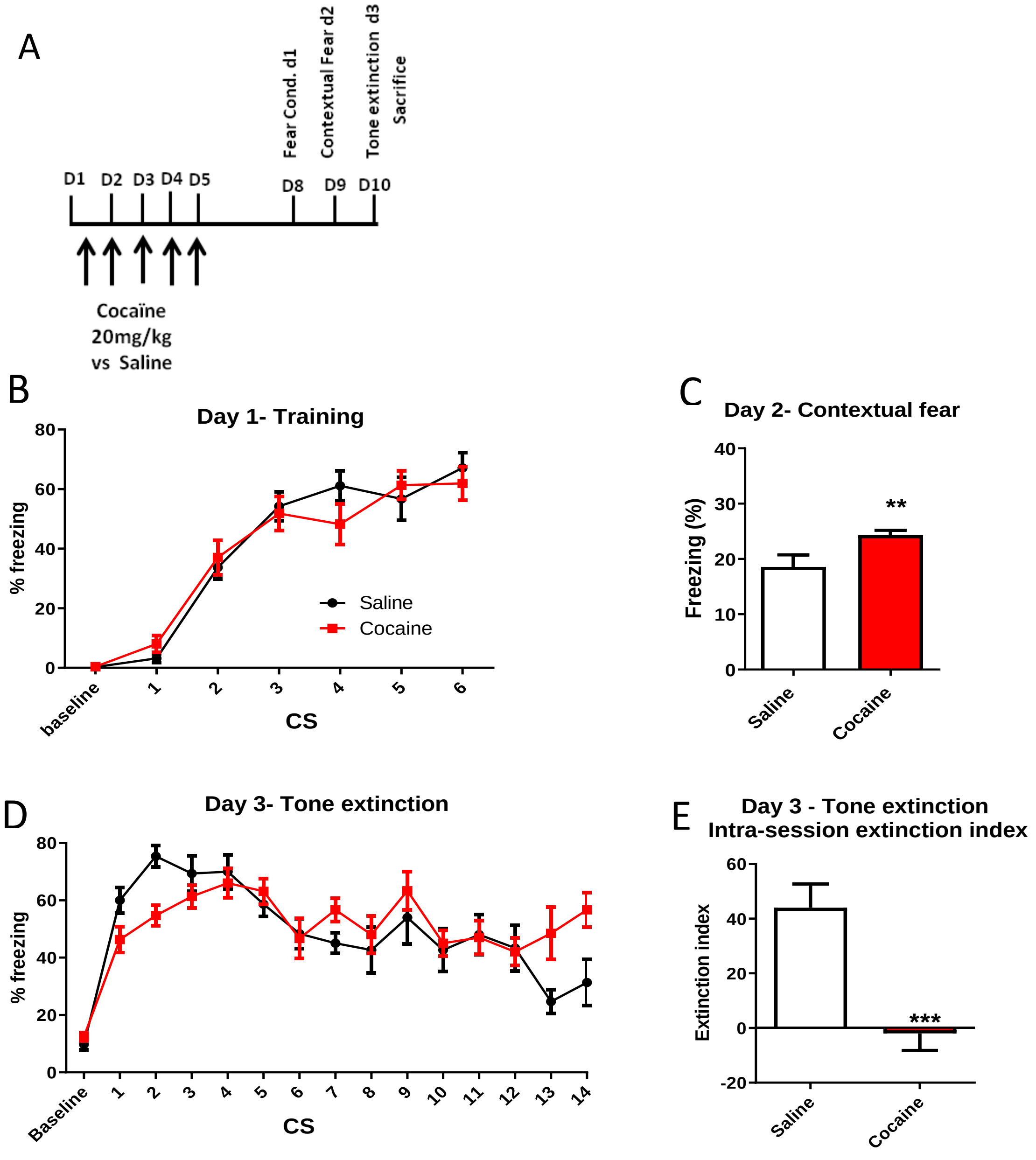

Figure S3: Subchronic exposure to cocaine increased contextual fear and decreased cued fear extinction memory in C57BL/6J mice. A. Experimental design**.** B. There was no effect of cocaine on fear acquisition in WT C57BL/6J mice. C. The following day, cocaine-treated mice exhibited increased contextual fear compared to saline-treated mice, $t(14.7) = 2.45$, $p < 0.01$. D. On Day 3, when mice were exposed to a different context, saline-treated mice and cocaine-treated mice displayed a different cued fear extinction profile, as indicated by the two-way ANOVA analysis [CS x genotype effect (F(13, 286)=2,87, p<0.001), CS effect

$F(13,286)=6.89$, $p<0.001$, no treatment effect, $F(1,22)=0.26$, $p=0.61$]. E. In contrast to saline-treated mice, cocaine-treated mice displayed a fear extinction deficit [(Day 3 freezing at CS2) − (Day 3 freezing at CS14), $t(22) = 4.31$, $p < 0.001$]. Cocaine (n=12) vs saline (n=12) ***p<0.001.
